# Comprehensive Analysis of Human Cytomegalovirus MicroRNA Expression during Lytic and Quiescent Infection

**DOI:** 10.1371/journal.pone.0088531

**Published:** 2014-02-12

**Authors:** Zhang-Zhou Shen, Xing Pan, Ling-Feng Miao, Han-Qing Ye, Stéphane Chavanas, Christian Davrinche, Michael McVoy, Min-Hua Luo

**Affiliations:** 1 State Key Laboratory of Virology, Wuhan Institute of Virology, Chinese Academy of Sciences, Wuhan, Hubei, China; 2 INSERM-U1043/CNRS-U5282/Paul Sabatier University, Toulouse, France; 3 Department of Pediatrics, Virginia Commonwealth University, School of Medicine, Richmond, Virginia, United States of America; Temple University School of Medicine, United States of America

## Abstract

**Background:**

Human cytomegalovirus (HCMV) encodes microRNAs (miRNAs) that function as post-transcriptional regulators of gene expression during lytic infection in permissive cells. Some miRNAs have been shown to suppress virus replication, which could help HCMV to establish or maintain latent infection. However, HCMV miRNA expression has not been comprehensively examined and compared using cell culture systems representing permissive (lytic) and semi-permissive vs. non-permissive (latent-like) infection.

**Methods:**

Viral miRNAs levels and expression kinetics during HCMV infection were determined by miRNA-specific stem-loop RT-PCR. HCMV infected THP-1 (non-permissive), differentiated THP-1 (d-THP-1, semi-permissive) and human embryo lung fibroblasts (HELs, fully-permissive) were examined. The impact of selected miRNAs on HCMV infection (gene expression, genome replication and virus release) was determined by Western blotting, RT-PCR, qPCR, and plaque assay.

**Results:**

Abundant expression of 15 HCMV miRNAs was observed during lytic infection in HELs; highest peak inductions (11- to 1502-fold) occurred at 48 hpi. In d-THP-1s, fourteen mRNAs were detected with moderate induction (3- to 288-fold), but kinetics of expression was generally delayed for 24 h relative to HELs. In contrast, only three miRNAs were induced to low levels (3- to 4-fold) during quiescent infection in THP-1s. Interestingly, miR-UL70-3p was poorly induced in HEL (1.5-fold), moderately in THP-1s (4-fold), and strongly (58-fold) in d-THP-1s, suggesting a potentially specific role for miR-UL70-3p in THP-1s and d-THP-1s. MiR-US33, -UL22A and -UL70 were further evaluated for their impact on HCMV replication in HELs. Ectopic expression of miR-UL22A and miR-UL70 did not affect HCMV replication in HELs, whereas miR-US33 inhibited HCMV replication and reduced levels of HCMV *US29* mRNA, confirming that *US29* is a target of miR-US33.

**Conclusions:**

Viral miRNA expression kinetics differs between permissive, semi-permissive and quiescent infections, and miR-US33 down-regulates HCMV replication. These results suggest that miR-US33 may function to impair entry into lytic replication and hence promote establishment of latency.

## Introduction

Human cytomegalovirus (HCMV) is a ubiquitous pathogen infecting 50% to 90% of the population worldwide, with an extremely high prevalence (>90%) in China. HCMV infection is not believed to be deleterious to immunocompetent individuals. However, it can cause serious, often life-threatening complications in immunocompromised individuals, including solid organ and cell transplant recipients, AIDS patients, and patients suffering from late stage cancers (reviewed by Mercorelli [1]). Above all, congenital HCMV infection of immunologically immature fetuses is the most common viral cause of birth defects, affecting 0.1–0.3% of newborns [2], [3].

HCMV establishes life-long latency following primary infection. Latency is a shared feature of *Herpesviridae* and for viruses in the alpha- and gamma-herpesvirus subfamilies is associated with expression of viral microRNAs (miRNA) (reviewed in [4]). MiRNAs are a class of non-coding RNAs of about 20–22 nucleotides (nt) in length. More than 10,000 miRNAs have been identified in a variety of organisms [5]. They participate in developmental processes (hematopoiesis, organogenesis, cell proliferation, differentiation and apoptosis), regulation of virus infection and anti-viral immune responses [4].

Viral miRNAs play important roles in regulation of virus infection by interacting with virus genes or regulating host genes to create a favorable cellular environment for virus replication [6]. Viral miRNAs were first identified in Epstein-Barr virus (EBV), a gamma-subfamily member of *Herpesviridae* [7]. To date over 230 viral miRNAs have been identified; most of which are encoded by herpesviruses. EBV encodes 25, Kaposi's sarcoma-associated herpesvirus (KSHV) encodes 12, murine cytomegalovirus (MCMV) encodes 18, and HCMV expresses 16 miRNAs [4], [8].

During latency the lytic viral replication cycle is repressed such that viral DNA is present but no infectious virus is produced. Upon certain external stimulations latent virus can be reactivated to lytic replication. The molecular mechanisms that govern establishment, maintenance, and reactivation from latency are poorly understood. MiR-BART2 encoded by EBV inhibits the lytic viral gene *BALF5* and thus may play an important role in maintenance of EBV latency [9]. Similarly, KSHV-encoded miR-9 inhibits expression of the viral protein RTA and miR-K1 targets IκBα to activate the NF-κB pathway, which in turn prevents lytic infection of KSHV and promotes latency [10], [11]. Viral miRNAs also regulate host immune responses and mediate viral immune evasion. For example, HCMV miR-UL112-1, EBV miR-BART2-5p, and KSHV miR-K12-7 help to protect infected cells from natural killer (NK) cell recognition by suppressing expression of major histocompatibility complex class I-related chain B (MICB), which mediates NK cell recognition via NKG2D [12], [13].

The functions of some HCMV miRNAs have been characterized. MiR-UL112-1 targets UL123 (IE1), UL112/113 and UL120/121, which are important for HCMV lytic infection [14]. HCMV miR-US25-1 and miR-US25-2 indirectly regulate viral replication, and miR-US25-1 inhibits expression of cytokines and cellular factors involved in signaling and cell cycle [15].

In the present work expression and kinetics of 16 mature HCMV miRNAs were examined in different cell culture systems representing permissive, semi-permissive, and quiescent/latent-like infections. THP-1 acute monocytic leukemia cells [16] were used to model quiescent infection. Infection of these cells results in maintenance of the HCMV genome without progression to the lytic replication pathway [17]–[23]. Viral gene expression is largely repressed during quiescent infection, but expression of lytic genes and viral DNA amplification can be induced by phorbol-ester treatment [18], [19], [24], which drives differentiation of THP-1 cells into macrophages [25]. THP-1 cells differentiated prior to infection (d-THP-1) were used as a model of semi-permissive infection as 10 - 40% of these cells become productively infected [20], [23], [26]. Human embryonic lung fibroblasts (HELs) were used to model highly efficient fully permissive lytic replication. Most of the HCMV miRNAs were abundantly expressed during permissive and semi-permissive replication and repressed during quiescent infection. One miRNA was selectively induced in THP-1/d-THP-1 cells and not in HELs. HCMV miRNAs were grouped in three groups based on kinetic patterns of expression in HELs. Three previously uncharacterized miRNAs (miR-UL22A, miR-UL70 and miR-US33), each representing one group, were further examined to determine their impact on viral replication. At low multiplicity of infection (MOI) miR-US33 down regulated viral gene expression, inhibited viral genome replication, and reduced infectious virus yield. These results suggest that miR-US33 is a player in regulating HCMV lytic infection.

## Materials and Methods

### Ethics statement

The Wuhan Institute of Virology Institutional Review Board approved the isolation of primary human embryonic lung fibroblasts from postmortem fetal embryo tissue and waived the need for consent. The original source of the postmortem fetal embryo tissue was the Zhongnan Hospital.

### Cells and cell culture

Primary HELs (maintained in our laboratory, [27]), THP-1 cells (ATCC- TIB202), and embryonic kidney 293T/17 cells (ATCC-CRL11268) were cultured in Eagle's minimal essential medium (MEM, Gibco BRL), RPMI 1640 medium (Gibco BRL) and Dulbecco's modified Eagle's medium (DMEM, Gibco BRL), respectively. All media were supplemented with 10% fetal bovine serum, 100 units/ml penicillin, 100 µg/ml streptomycin and 2 mM L-glutamine (Gibco BRL). THP-1 cells were differentiated (d-THP-1) by treatment with phorbol 12-myristate 13-acetate (PMA, 12.5 ng/ml) and hydrocortisone (1.8 µg/ml) (Sigma) for 24 hour (h) prior to HCMV infection as described [26]. Cells were maintained at 37°C in a humidified atmosphere containing 5% CO_2_.

### Plasmid construction and lentiviruses production

Lentivirus expression system, including plasmids pCDH-CMV-MCS-EF1-copGFP and pML-delta8.9 and pVSV-G (System Biosciences, CA, USA), was used to ectopically express HCMV-encoded miRNAs. Sequences of HCMV-encoded pre-miRNAs were obtained from miRBase/Release 20 (http://www.mirbase.org/) and PCR-amplified using HCMV DNA (strain Towne) as the template. Primers and product sizes are listed in Table 1. PCR products were cloned into EcoRI/BamHI-digested pCDH-CMV-MCS-EF1-copGFP to make plasmids pCDH-miR-UL22A, pCDH-miR-UL70 and pCDH-miR-US33 containing miR-UL22A, miR-UL70, miR-US33 inserts, respectively. Lentiviruses were generated by co-transfecting 15 µg of pCDH-miR-UL22A, pCDH-miR-UL70, or pCDH-miR-US33 with 6 µg of pVSV-G (encodes vesicular stomatitis virus G protein) and 12 µg pML-delta8.9 (encodes HIV structural proteins) into 293T cells using calcium phosphate as described [28]. At 48 h post transfection the supernatants containing lentiviruses were collected, clarified of cell debris by centrifugation (1500 rpm, 10 min×2), filtered through a 0.45 µm membranes, and stored at −80°C. Lentivirus stocks were titered by counting copGFP-positive cells as described [29].

**Table 1:** Primers used for cloning HCMV pre-miRNAs.

Primer names	Primers sequence		Product size
miR-UL22AF	CCGGAATTCTCTTCCCATAGCCTGTCTAA		
miR-UL22AR	CGCGGATCCGCCGCAGCATCCCGCATA	100 bp	
miR-UL70F	CGCGGATCCGAGAGCACGGGCGTGCGGAA		
miR-UL70R	CCGGAATTCGCCGTGAACAACGAAACGCT	140 bp	
miR-US33F	CGCGGATCCGACGTAGGAAAGGATCATGT		
miR-US33R	CCGGAATTCTAAGACGTGTCATTGTTGTC	200 bp	

### Virus and infection

HCMV Towne strain (ATCC-VR977) was propagated and titered as described previously [30]. For kinetic studies of miRNA expression, HELs were infected with HCMV at a multiplicity of infection (MOI) of 5. After 3 h the culture medium was replaced with fresh medium. THP-1 and d-THP-1 cells were infected in parallel at an MOI of 10.

HELs were transduced with lentiviruses lacking an insert (vector control) or expressing HCMV miRNAs at an MOI of 10. After confirmation of miRNA expression by RT-PCR at 48 h after transduction (described below), transduced HELs were infected with HCMV at MOIs of 0.01, 0.1, 1, or 5. Cells were harvested for RNA, DNA, and Western blotting studies at 24, 48 and 72 h post infection (hpi) and supernatants were collected and titered for infectious HCMV at 96 and 144 hpi.

### RT-PCR

HCMV-encoded miRNAs were detected by stem-loop RT-PCR. Total RNA was isolated from HCMV-infected cells using Trizol Reagent (TaKaRa) and DNA was removed using Recombinant DNase I (TaKaRa). One µg of each RNA sample was reverse transcribed with RevertAid™ H Minus First Strand cDNA Synthesis Kit (Fermentas) using miRNA-specific stem-loop RT primers (Table 2) as described previously [31]-[33]. RT reaction products were quantified by quantitative PCR (qPCR) using All-in-One™ qPCR Mix (GeneCopoeia) with a CFX Connect™ Real-Time System (BIO-RAD). 20 µl PCR reactions contained 2 µl RT reaction product, 10 µl 2×qPCR Mix, and 250 nM forward and reverse primers (Table 2). Reactions were denatured at 95°C for 3 min, followed by 40 two-step cycles of 95°C for 10 s and 60°C for 30 s. Expression of HCMV-US29 was assessed by qRT-PCR with specific F primer (5′-CGACGAGACAACAATGAC3′) and R primer (5′-AATTGACGGTCCACTGAG3′) as described above. Expression levels of 5sRNA were measured to confirm consistency of RNA extraction and amplification between samples.

**Table 2:** Primers used for quantitation of viral miRNA by stem-loop RT-PCR.

Primer names	Primer sequences	size
RT (miRNAs)		
5S rRNA	CTCAACTGGTGTCGTGGAGTCGGCAATTCAGTTGAGAAAGCCTA	
UL22A-5p	CTCAACTGGTGTCGTGGAGTCGGCAATTCAGTTGAGTCTCACGG	
UL22A-3p	CTCAACTGGTGTCGTGGAGTCGGCAATTCAGTTGAGCTACAAAC	
UL36-5p	CTCAACTGGTGTCGTGGAGTCGGCAATTCAGTTGAGTCTTTCCA	
UL36-3p	CTCAACTGGTGTCGTGGAGTCGGCAATTCAGTTGAGGCACGTTG	
UL70-5p	CTCAACTGGTGTCGTGGAGTCGGCAATTCAGTTGAGTCTGGACG	
UL70-3p	CTCAACTGGTGTCGTGGAGTCGGCAATTCAGTTGAGCCGCGCGC	
UL112	CTCAACTGGTGTCGTGGAGTCGGCAATTCAGTTGAGAGCCTGGA	
US4	CTCAACTGGTGTCGTGGAGTCGGCAATTCAGTTGAGATCCCCCT	
US5-1	CTCAACTGGTGTCGTGGAGTCGGCAATTCAGTTGAGACGCTCTC	
US5-2	CTCAACTGGTGTCGTGGAGTCGGCAATTCAGTTGAGGACATCGT	
US25-1-5p	CTCAACTGGTGTCGTGGAGTCGGCAATTCAGTTGAGGGTCCGAG	
US25-1-3p	CTCAACTGGTGTCGTGGAGTCGGAATTCAGTTGAGGAGAACCG	
US25-2-5p	CTCAACTGGTGTCGTGGAGTCGGCAATTCAGTTGAGTCATCCAC	
US25-2-3p	CTCAACTGGTGTCGTGGAGTCGGCAATTCAGTTGAGCCGCGGGA	
US33-5p	CTCAACTGGTGTCGTGGAGTCGGCAATTCAGTTGAGCGCCCACG	
US33-3p	CTCAACTGGTGTCGTGGAGTCGGCAATTCAGTTGAGTGGATGTG	
PCR (miRNAs)		
5S rRNA	GTCTACGGCCATACCACCCTGAAC	157 bp
UL22A-5p	TAACTAGCCTTCCCG	56 bp
UL22A-3p	TCACCAGAATGCTAGT	58 bp
UL36-5p	TCGTTGAAGACACCTG	58 bp
UL36-3p	TTTCCAGGTGTTTTCA	58 bp
UL70-5p	TGCGTCTCGGCCTCGT	57 bp
UL70-3p	GGGGATGGGCTGGCGC	56 bp
UL112	AAGTGACGGTGAGATCCA	58 bp
US4	CGACATGGACGTGCAG	58 bp
US5-1	TGACAAGCCTGACGAG	57 bp
US5-2	TTATGATAGGTGTGAC	58 bp
US25-1-5p	AACCGCTCAGTGGCTC	57 bp
US25-1-3p	TCCGAACGCTAGGTCG	58 bp
US25-2-5p	AGCGGTCTGTTCAGGT	58 bp
US25-2-3p	ATCCACTTGGAGAGCT	59 bp
US33-5p	GATTGTGCCCGGACCGTG	58 bp
US33-3p	TCACGGTCCGAGCAC	56 bp
Universal R	CTCAACTGGTGTCGTGGA	

### qPCR

DNA was extracted from cell pellets using TIANamp Genomic DNA Kit (TIANGEN) and adjusted to 10 ng/µl. HCMV DNA was detected using primers from the IE1 gene locus (F primer 5′AACTCAGCCTTCCCTAAGACCA3′, R primer 5′ CAGCACCCGACAGAACTCAC 3′). Amplification of GAPDH sequences using primers F 5′ GTTTACATGTTCCAATATGA 3′ and R 5′ TCCTGGAAGATGGTGATGGG served as an internal reference. 20 µl qPCR reactions contained 2 µl (20 ng) DNA, 50 nM forward and reverse primers, and 10 µl 2× iTaqTMSYBR Green Supermix (BIO-RAD). Reactions were denatured at 95°C for 5 min, followed by 40 cycles of 95°C for 10 s and 54°C for 20 s using a CFX Connect™ Real-Time Sysem (BIO-RAD).

### Western blotting

Cell lysates were prepared as described previously [30]. Protein concentrations were quantified using the BCA assay (Beyotime) and equal amounts of protein (50 µg) were separated by SDS-PAGE using 8% polyacrylamide gels. Proteins were transferred to Immobilon®-P membranes (IPVH00010, Millipore). Detection of proteins was described previously [30]. Primary antibodies included mouse monoclonals Ch16.0 (anti-HCMV IE1/2; Virusys), Ch13.0 (anti-HCMV UL44; Virusys), 27–156 (anti-HCMV glycoprotein B, a gift from William Britt, University of Alabama), and C4 (anti-actin; Santa Cruz Biotechnology). The secondary antibody was horseradish peroxidase-conjugated sheep anti-mouse IgG (GE healthcare).

### Luciferase assay

A luciferase reporter assay was constructed to assess miR-US33 regulation of *US29* expression. A 500-bp region of *US29* sequence was PCR amplified using primers 5′CGGGGTACCGCTCTACAGTGGGTGGTGGT3′ and 5′CCGCTCGAGAAGCGTTGCCGTAGCTGGCG3′ and cloned into a modified pGL3 plasmid, pGL3cM, that contains a firefly luciferase reporter gene (Promega, Madison, USA) to make plasmid pGL3-Luc-US29. β-galactosidase (β-gal) expression from plasmid pCMV-SPORT-β-gal (kind gift from J Melendez at the College of Nanoscale Sciences and Engineering, SUNY, Albany, USA) was used as a transfection control. pGL3-Luc-US29 and pCMV SPORT-β-gal were cotransfected into 293T cells along with either pCDH-miR-US33 or the pCDH-copGFP empty vector control using calcium phosphate as described [34]. Cells were harvested at 48 h post transfection, luciferase activities were measured using pGL3 Luciferase Reporter Vectors kit (Promega), and β-gal activities were measured using β-Galactosidase Enzyme Assay System with Reporter Lysis Buffer kit (Promega). Signals were quantitated using a Modulus TM II microplate multimode reader.

### Statistical analyses

All reactions were run in triplicate for each experiment. Three independent experiments were performed. Data were analyzed by One-way ANOVA. Results reported are means ± standard deviations (SD). Differences were considered to be significant when P values were less than 0.05.

## Results

### HCMV miRNA expression during permissive replication in HELs

**Figure 1:**
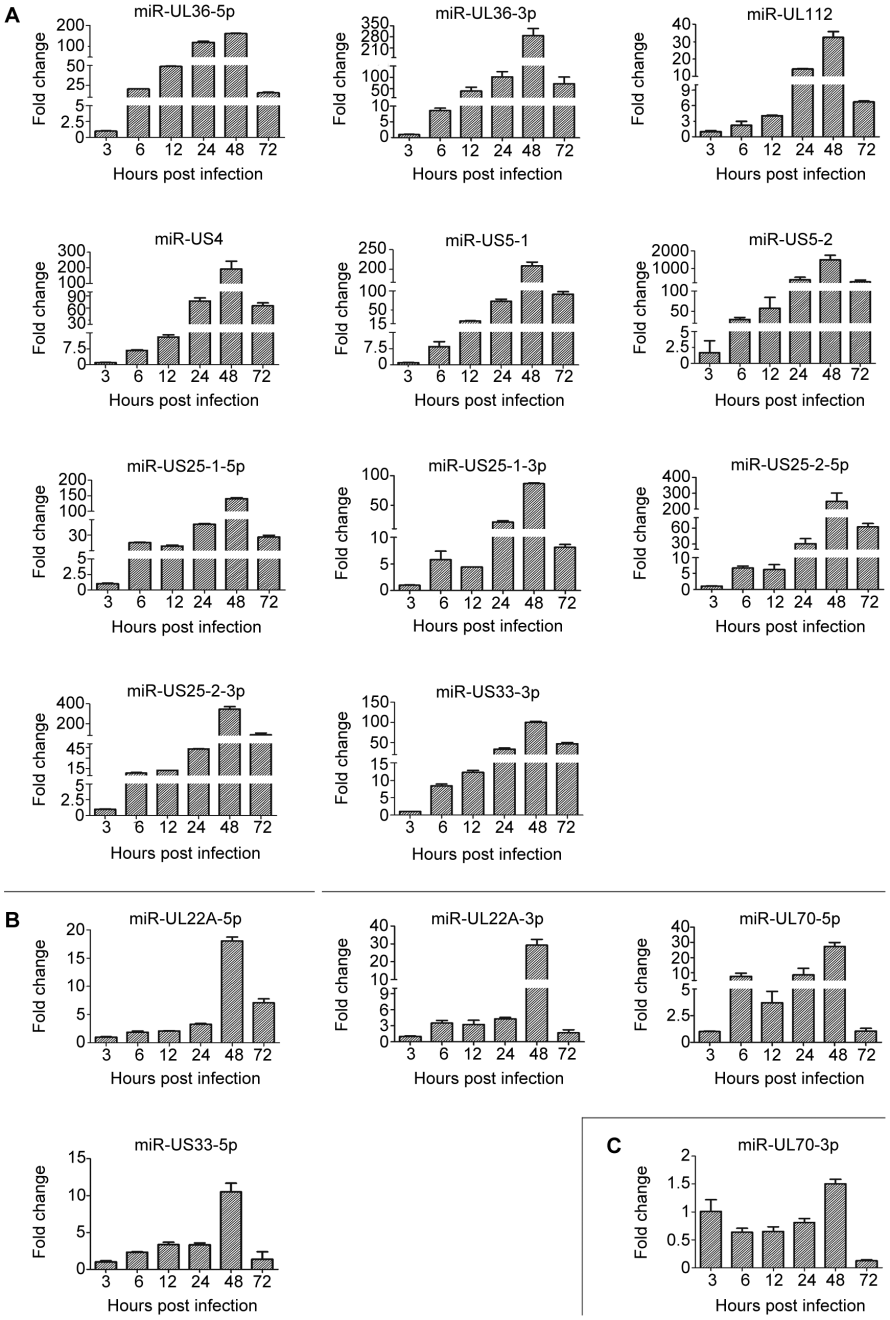
Expression kinetics of HCMV miRNAs during lytic replication in HELs. HELs were infected with HCMV strain Towne at an MOI of 5 and intracellular HCMV miRNAs were quantitated by stem-loop RT-PCR at the indicated times post infection. Results indicate fold-changes relative to levels measured at 3 hpi. HCMV miRNAs were assigned to group 1 (A), group 2 (B) or group 3 (C) based on kinetic patterns of expression (see text for details).

Twenty three mature miRNAs encoded by HCMV have been reported, however, only 16 of the 23 mature miRNAs are in the database of miRBase/Release 20 [35]. To evaluate the expression kinetics of HCMV-encoded miRNAs during productive infection in fully-permissive cells, HELs were infected with HCMV (Towne) at an MOI of 5 and RNA was isolated at different time points post-infection. Levels of the 16 HCMV miRNAs described in miRBase/Release 20 were measured by stem-loop RT followed by qPCR. Relative expression levels were expressed as fold changes relative to levels at 3 hpi. All 16 miRNAs increased as early as 6 hpi and reached maximum levels at 48 hpi followed by either steep or gradual declines at 72 hpi (Fig. 1).

Based on patterns of expression, HCMV miRNAs could be classified into three groups. Group 1 miRNAs (11 miRNAs: miR-UL36-5/3p, -UL112, -US4, -US5-1, -US5-2, -US25-1-5/3p, -US25-2-5/3p and -US33-3p) increased gradually and reached high expression levels (>30-fold induction) at 48 hpi, then declined somewhat but remained high (>8-fold) at 72 hpi, when progeny virus production is vigorous (Fig. 1A). Group 2 miRNAs (4 miRNAs: miR-UL22A-5/3p, -UL70-5p and -US33-5p) increased more slowly to reach moderate levels (<10-fold) before 48 hpi, then abruptly increased to 10- to 30-fold levels at 48 hpi, and decreased steeply to low levels at 72 hpi (Fig. 1B). The one miRNA in group 3 (miR-UL70-3p) showed no induction prior to 48 hpi, peaked at a low level (1.5-fold) at 48 hpi, and thereafter declined dramatically (Fig. 1C).

These results suggest that group 1 miRNAs may have functions that are important early in the HCMV replicative cycle, as significant levels are attained prior to the onset of DNA synthesis at ∼24 hpi. Group 2 and 3 miRNAs may have later functions as their levels do not rise until 48 hpi, when viral DNA synthesis is underway and progeny virus assembly has begun.

### HCMV miRNAs were repressed during quiescent infection in THP-1 cells

**Figure 2:**
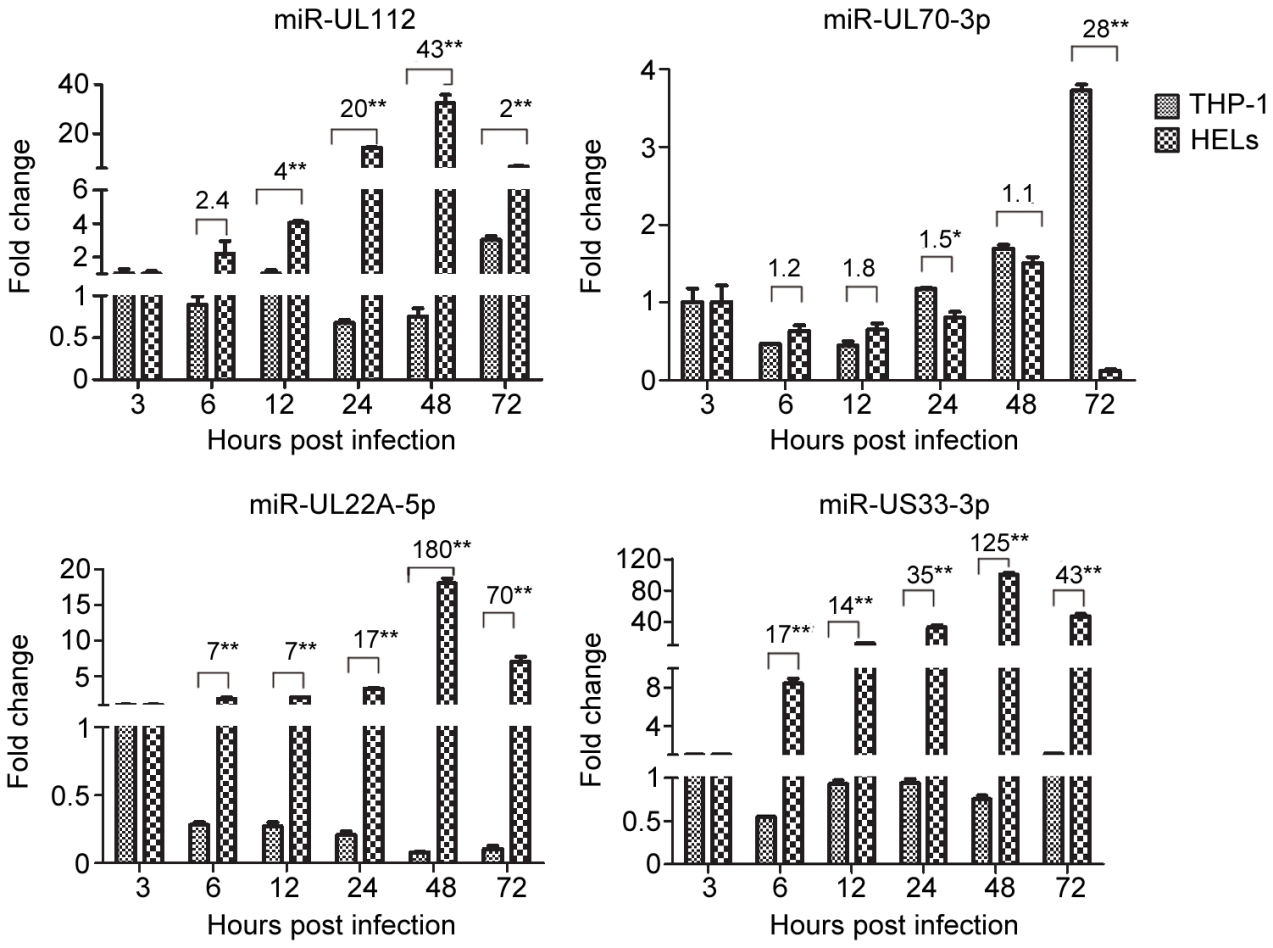
Comparison of miRNA expression in HCMV infected HEL and THP-1 cells. Expression kinetics for four HCMV miRNAs representing the three expression pattern groups are shown side-by-side to compare expression patterns in HELs vs. THP-1s (data are from experiments described in Figs. 1 and S1). The compared representatives of group 1 (miR-112 and miR-70-3p), group 2 (miR-22A-5p), and group 3 (miR-US33-3p) are shown. Note that HELs and THP-1 cells were infected with HCMV strain Towne at MOIs of 5 and 10, respectively. *P<0.05, **P<0.01.

To examine HCMV miRNA expression during quiescent infection, undifferentiated THP-1 monocytes were infected at an MOI of 10 and expression levels of the 16 HCMV miRNAs were determined at different hpi. Thirteen of the 16 miRNAs exhibited either little change or declined significantly after infection. This is in stark contrast to HELs in which these miRNAs increased 11- to 1502-fold. Three miRNAs (miR-UL70-3p, -UL112 and -US5-1) exhibited slight (3 to 4-fold) inductions, but only at 72 hpi (Fig. S1). The relative inductions of these three miRNAs in THP-1s differed significantly from those in HELs (*i.e.*, 3-fold vs. 33-fold for miR-UL112, 4-fold vs. 1.5-fold for miR-UL70-3p, and 3-fold vs. 209-fold for miR-US5-1). Differences between the two cell types in kinetics and induction levels for four representative miRNAs are illustrated in Fig. 2. In HELs miR-UL112 and miR-US33-3p (group 1) were rapidly induced, while miR-UL22A-5p (group 2) was delayed. However, by 48 hpi all three reached high peak levels (32-, 101-, and 19-fold, respectively). In contrast, all three miRNAs declined in THP-1 cells after 3 hpi and either remained very low (miR-UL22A-5p) or increased slightly (mi-UL112 and -US33-3p) at 72 hpi. Remarkably, kinetics of miR-UL70-3p were quite similar in the two cell types until 72 hpi when there was a 4-fold increase in THP-1 cells and a dramatic decline in HELs (Fig. 2).

These results suggest that, similar to lytic gene transcription, most HCMV miRNAs are repressed in THP-1 cells. MiR-UL70-3p appears to be uniquely expressed in THP-1s but its late time of induction would seem to preclude a role in establishing a quiescent state.

### Differentiation resulted in increased induction of many but not all miRNAs during semi-permissive infection in d-THP-1 cells

**Figure 3:**
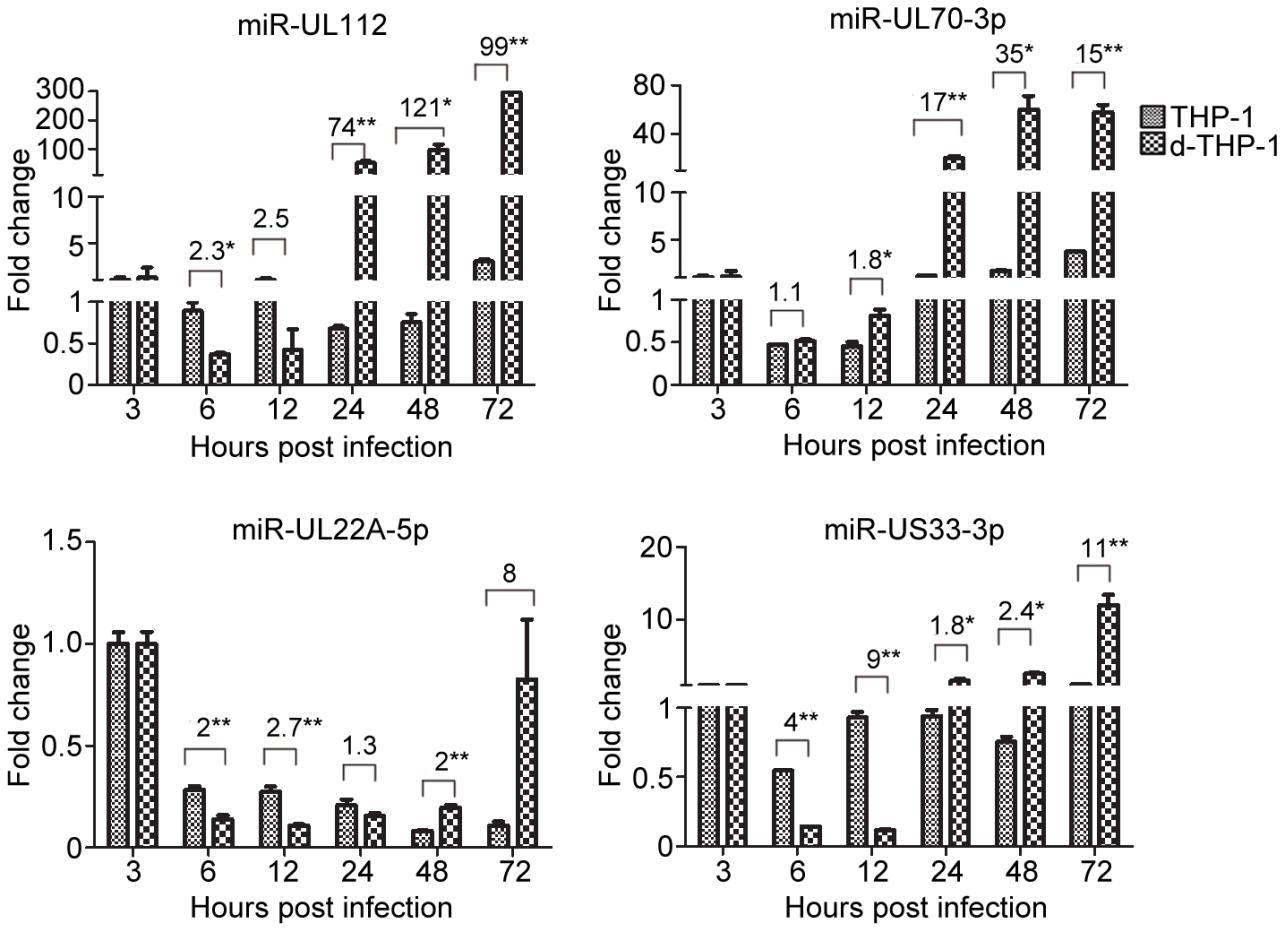
Expression comparison of representatives of miRNAs in THP-1 and d-THP-1 cells. The THP-1 and d-THP-1 cells were infected with Towne at an MOI of 10, and cells were harvest at the indicated times post infection, respectively. Expression kinetics for four HCMV miRNAs (miR-112 and miR-70-3p, miR-22A-5p, and miR-US33-3p) is shown side-by-side to compare expression patterns in THP-1 vs. d-THP-1(data are from experiments described in Fig S1 and S2). The different fold change of time points were shown above, **P<0.01, *P<0.05.

To determine how differentiation to a semi-permissive state alters the expression of HCMV miRNAs, THP-1 cells were differentiated by culture for 24 h in the presence of PMA and hydrocortisone prior to HCMV infection. The resulting d-THP-1 cells were infected at an MOI of 10 and expression levels of the 16 HCMV miRNAs were determined at different hpi. Differentiation profoundly affected patterns and levels of miRNA expression in d-THP-1s relative to THP-1s. Thirteen of the 15 miRNAs (except miR-UL70-3p) that were strongly induced during HEL infection (but not induced in THP-1 cells) exhibited induction increases as a result of differentiation (Fig. S2). Of these, three were increased over THP-1 levels but inductions remained relatively low (<4-fold; miR-US25-2-5p, -UL22A-5p, and -UL22A-3p), four were significantly induced (>7-fold) but not to levels seen in HELs (miR-UL36-5p, -US5-1, -US25-2-3p, -US33-3p), four increased to levels very similar to those in HELs (miR-US4, -US25-1-5p, -US25-1-3p, and -US70-5p), and two reached levels significantly higher than those in HELs (miR-UL112 and -US33-5p) (Table S1). In general miRNA expression was delayed approximately 24 h in d-THP-1s vs. HELs. One exception was miR-US33-5p, which was induced to higher levels 24 h earlier in d-THP-1 cells vs. HELs. The other exception was miR-UL70-3p, which was basically not induced in HELs, but dramatically induced to higher levels from 24 hpi on and maintained higher (Fig 3, Fig S2 and Table S1).

Differences between THP-1 and d-THP-1 cells in kinetics and induction levels for four representative miRNAs are illustrated in Fig. 3. MiR-UL112 and miR-UL70-3p were essentially uninduced in THP-1s but were induced dramatically in d-THP-1s from 24 hpi on. In contrast, miR-UL22A-5p was uninduced in both cell types while miR-US33-3p was moderately induced in d-THP-1s at 72 hpi.

These results suggest that a subset of HCMV miRNAs are significantly expressed during lytic infection independent of cell type, while high expression levels of other miRNAs are unique to HELs or d-THP-1s. This suggests that differences in HCMV miRNA expression between THP-1, d-THP-1, and HEL cells cannot be simply explained by different percentages of cells entering quiescent vs. lytic infection in each cell type. The exclusive induction of miR-UL70-3p in d-THP-1s indicates that miR-UL70-3p induction may be THP-1 cell specific and cell differentiation associated.

### HCMV miR-US33 inhibited HCMV replication

**Figure 4:**
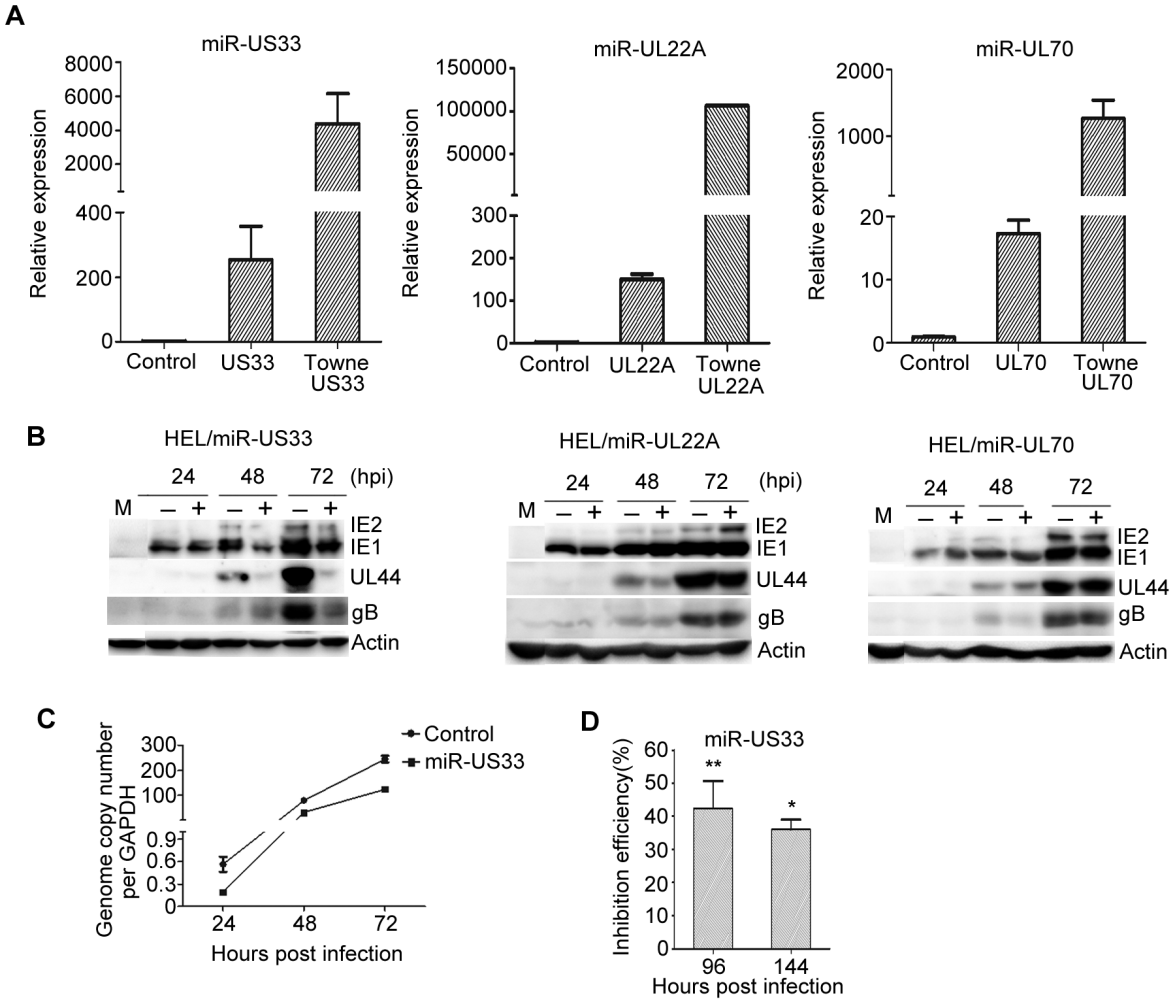
Impact of ectopic expression of miRNAs on HCMV replication. (A) HCMV miRNA levels were quantitated by stem-loop RT-PCR in HEL cells 48 h after transduction with empty vector lentivirus (control) or with lentiviruses engineered to express miR-UL22A, miR-UL70, miR-US33, or 48 hpi of HELs with HCMV strain Towne (MOI = 5). Results represent fold-differences for each miRNA relative to control. (B) Levels of IE1, IE2, UL44, or gB were determined by western blotting. Actin serves as a loading control. Control lentivirus (−) or lentiviruses expressing according miRNAs (+) are indicated. (C) HELs were infected with HCMV strain Towne at an MOI of 0.01 48 h after transduction with control lentivirus or lentivirus expressing miR-US33. At the times indicated post infection HCMV genome copy numbers were determined by qPCR. (D) HELs were infected with HCMV strain Towne at an MOI of 0.01 48 h after transduction with control lentivirus or lentivirus expressing miR-US33 Infectious virus titers in supernatants at 96 and 144 hpi were determined. Inhibition efficiencies are % changes in virus titers in medium from HCMV-infected miR-US33-transduced cells vs. control-transduced cells. **P<0.01, *P<0.05.

Three previously uncharacterized HCMV miRNAs, miR-US33, miR-UL22A and miR-UL70, were selected for further study based on changes in induction upon differentiation. HELs were transduced with lentiviruses expressing pre-miR-US33, pre-miR-UL22A, pre-miR-UL70, or empty vector control. Each miRNA was measured by stem-loop RT/qPCR at 48 h after transduction. The signal from miR-US33-transduced cells was 254-fold above that of empty vector-transduced cells, and those for miR-UL22A and miR-UL70 were 151-fold and 17-fold, respectively. However, transduced levels of miR-US33, miR-UL22A and miR-UL70 were 4375-, 106300- and 1267-fold lower, respectively, than levels induced 48 h post HCMV infection of untransduced HELs (Fig. 4A). Hence, ectopical expression of these miRNAs through lentivral transduction may only have been significant prior to 48 h post HCMV infection as by this time HCMV-encoded miRNA expression far exceeded lentiviral-mediated expression.

Transduced HELs were then infected with HCMV at MOIs of 0.01, 0.1, 1 or 5. Expression of representative immediate early (IE1/IE2), early (UL44), and late (glycoprotein B, gB) viral proteins was assessed by Western blotting, viral DNA replication was measured by qPCR, and virus yield was determined by titering infectious virus released into culture supernatants. Transduction of miR-UL22A-5p and miR-UL70-5p had no apparent effects on HCMV infection at all the MOIs tested, and miR-US33 had no effects at MOIs of 0.1, 1 or 5 (Fig. 4B, data not shown). However, at MOI = 0.01 levels of IE1/IE2, UL44, and gB were significantly reduced in miR-US33-transduced cells (Fig. 4B). Viral DNA replication was also reduced from 0.6 to 0.2 copies/cell at 24 hpi (P = 0.022), from 79 to 30 copies/cell at 48 hpi (P = 0.001), and from 245 to 123 copies/cell at 72 hpi (P = 0.001) (Fig. 4C). Finally, miR-US33 transduction reduced the amounts of infectious virus in the culture supernatants from 3.4×10^3^ to 1.9×10^3^ pfu/ml at 96 hpi (P = 0.0001) and from 8.6×10^5^ to 3.1×10^5^ pfu/ml at 144 hpi (P = 0.012) (Fig. 4D).

### MiR-US33 repressed expression of its target HCMV US29

**Figure 5:**
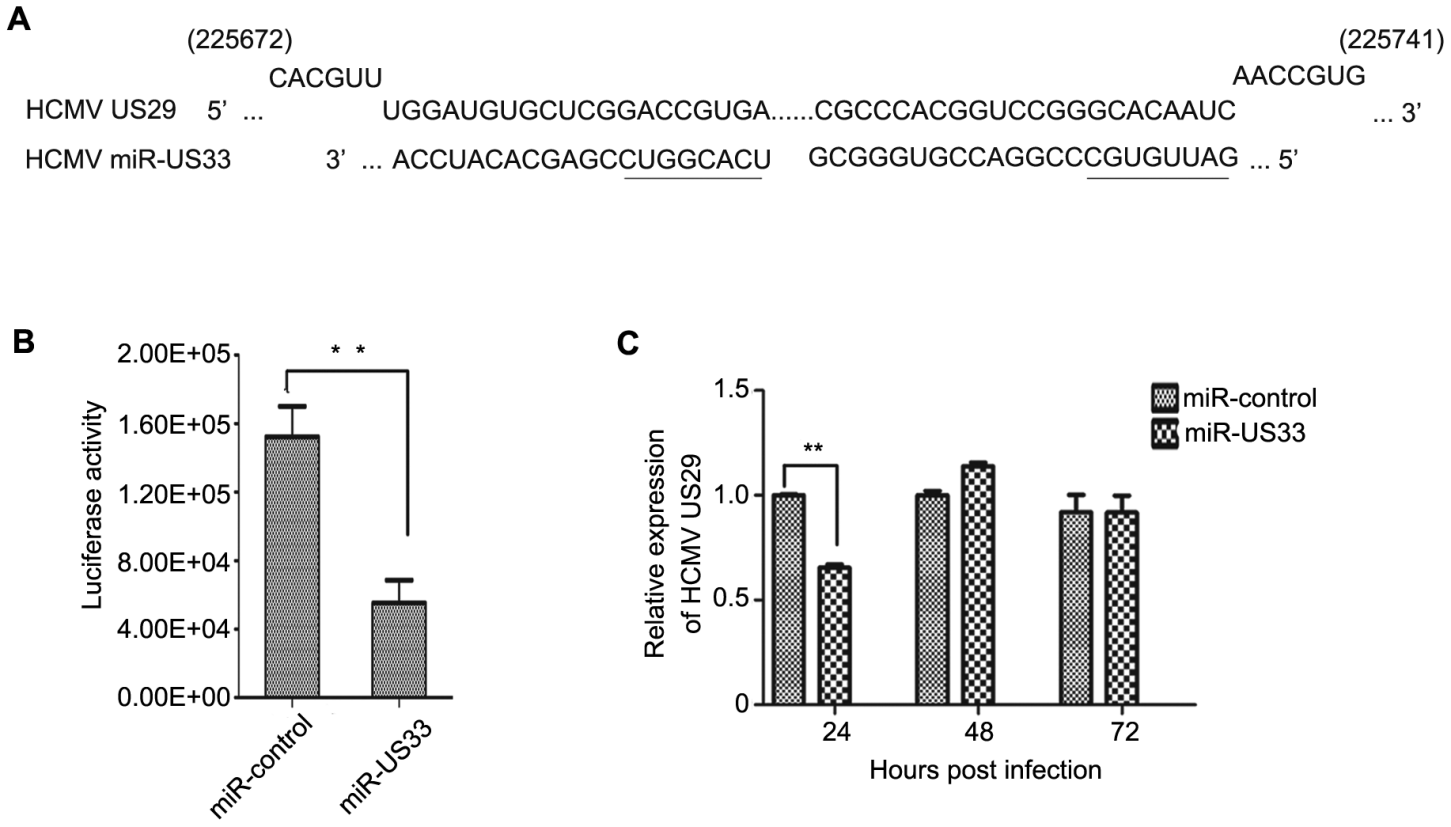
Down-regulation of HCMV US29 by miR-US33. (A) Complementarity of miR-US33 with its putative target sequence in *US29*; the miR-US33 “seed” sequence is underlined. (B) *US29* sequences are a target of miR-US33. A plasmid expressing luciferase from a transcript containing the putative miR-US33 target sequence from *US29* was co-transfected into 293 cells with a plasmid expressing miR-US33 or an empty vector control plasmid. Each transfection also included a β-gal-expressing plasmid to normalize transfection efficiencies. Luciferase and β-gal activities were measured 48 h post transfection and β-gal activities were used to normalize the luciferase activities. (C) Regulation of *US29* mRNA by miR-US33 during HCMV infection. HELs were infected with HCMV strain Towne at an MOI of 0.01 48 h after transduction with control lentivirus or lentivirus expressing miR-US33 and at the indicated hpi *US29* mRNA levels were quantitated by RT-PCR. **P<0.01.

Most miRNAs contain short (2–8 nt) ‘seed’ regions that can base pair with target mRNAs to form exactly matched 6–7 nt regions of double-stranded RNA [36]. Sequence alignments identified HCMV *US29* as a predicted target for miR-US33-3p [37]–[39] (Fig. 5A). A luciferase reporter assay was devised to determine if this *US29* sequence functions as a target for miR-US33. The predicted *US29* target sequences were inserted into the 3′ untranslated region of a mRNA encoding luciferase that was expressed from a plasmid by transient transfection. pCDH-miR-US33 or the pCDH-copGFP was cotransfected with the luciferease-*US29* reporter plasmid and a control plasmid expressing β-gal into 293T/17 cells. Luciferase activities were normalized to β-gal activities measured in the same cells 48 h post transfection. Cells expressing miR-US33 had 2.6-fold lower luciferase activity compared to those transduced with empty vector (P = 0.002) (Fig. 5B).

To determine whether *US29* transcripts are affected by miR-US33 during HCMV infection, HELs transduced with miR-US33-expressing or vector control lentiviruses were infected with HCMV (MOI = 0.01) and *US29* mRNA levels were quantitated by qRT-PCR. *US29* transcript levels were reduced by nearly half at 24 hpi (P = 0.002), confirming that miR-US33 can down-regulate *US29* mRNA in the context of HCMV infection (Fig. 5C). The inhibition of *US29* transcription became insignificant with the progress of HCMV infection at 48 and 72 hpi. The inhibition effect was probably masked by other viral proteins after the viral replication initiation.

## Discussion

Following primary infection HCMV establishes a latent/persistent infection characterized by latency with periodic or sporadic reactivation [40], [41]. Primary infection and reactivation result in serious, sometimes life-threatening complications in individuals with impaired or immature immune systems, including AIDS patients, transplant recipients, and fetuses infected *in utero* [1], [42]–[44].

During latency, expression of viral genes associated with lytic replication is repressed. In HCMV, robust expression of IE1/2 proteins through active transcription from the major immediate early promoter (MIEP) is crucial for driving cells forward in the lytic replication cycle. Conversely, repression of IE1/2 expression may promote establishment of latency in some cell types or abortive infection in others. During natural latency the MIEP is associated with markers of repressed chromatin and repressive chromatinization occurs rapidly after experimental infection in cell culture models of latency [17], [45]–[47]. Repressive chromatinization is thought to occur by default upon delivery of the viral genome to the nucleus; to enter lytic replication the viral tegument protein pp71 must traffic to the nucleus where it acts to de-repress IE transcription by inducing degradation of Daxx [48]. In cell culture latency models, including THP-1s, de-repression fails because pp71 does not enter the nucleus [49]. Thus, in the early hours post infection the decision to enter a latent vs. lytic program depends on interplay of cellular and viral factors to activate or repress the MIEP. Additional mechanisms may influence or fine-tune the decision making process. Once committed to a lytic or latent program, complex mechanisms govern progression through the lytic cycle, and presumably, additionally complex but little understood mechanisms are needed to establish and maintain the latent state and to drive re-entry into the lytic pathway upon reactivation.

The immunologically inert nature of miRNAs makes viral miRNA-mediated mechanisms for governing latency intellectually attractive, and indeed, control of viral and host genes by viral miRNAs during latency has been demonstrated for several viruses in the alpha- and gamma-herpesvirus subfamilies (reviewed in [4]). Thus far HCMV miRNAs have only been characterized during lytic infection. While such studies have identified HCMV miRNAs that mediate immune evasion, modulate cell cycle regulation, and either up- or down-modulate lytic replication (recently reviewed in [8], [50]), expression of HCMV miRNAs has not as yet been evaluated during latency either *in vivo* or in experimental cell culture latency models.

In the present work expression and kinetics of 16 mature HCMV miRNAs were examined during lytic replication in fibroblasts (Fig. 1) and directly compared during quiescent infection of undifferentiated THP-1 monocytes (Fig. 2 and Fig. S1) and semi-permissive lytic replication in THP-1-derived macrophages (d-THP-1, Fig. 3 and Fig. S2). While infection in THP-1 is not considered a true model of HCMV latency by some investigators because reactivation to produce infectious progeny has not be clearly established. THP-1 infection has been used to model maintenance of viral genomes in a transcriptional quiescent state [17]–[21], [49], [51]–[54].

Fifteen of the 16 HCMV-encoded miRNAs were highly expressed during lytic infection in HELs, consistent with previous findings that HCMV miRNAs are responsible for up to 20% of the total miRNAs in infected cells [55]. While kinetic class was not determined for miRNAs using inhibitors of protein or viral DNA synthesis, the 11 HCMV miRNAs designated as group 1 were induced to significant levels by 6 hpi and reached high peak levels at 48 hpi, suggesting possible roles in modulating lytic replication during immediate early, early, or late phases of replication. Functions for several of these miRNAs have been reported. HCMV miR-UL112-1 down-regulates MICB to escape NK cell recognition and targets viral proteins IE1 and UL114 [13], [56], while miR-US25-1 and miR-US25-2 target cell cycle regulators such as cyclin E2 [15], [39].

The less efficient lytic replication that occurs in d-THP-1 cells exhibited similarly robust induction of six HCMV miRNAs, suggesting a common core of miRNAs associated with lytic replication in both cell types. Consistent with a general delay in lytic gene expression [26], HCMV miRNA expression in d-THP-1 cells was delayed approximately 24 h relative to HEL infection (Fig. S2). However, two miRNAs that were highly expressed during lytic infection in HELs (miR-UL36-3p and miR-US5-2) were not detected at all in d-THP-1 cells, while seven, including miR-UL36-5p and miR-US5-1, were significantly less induced compared to HELs (Fig. S2, Fig. 1). It is tempting to speculate that low or absent expression of some of these miRNAs contributes to inefficiency of replication in d-THP-1 cells. Consistent with this hypothesis, two recent reports suggest roles for miR-UL36 and miR-US5-2 in promoting efficient lytic replication. In one study miR-UL36 was shown to down regulate expression of the latency-associated protein UL138 and ectopic expression of miR-UL36 enhanced HCMV DNA synthesis during the early stages of replication in fibroblasts [57]. The second study confirmed that miR-US5-1 and -2 target and down-regulate US7, and found that viral mutants lacking miR-US5-1, miR-US5-2, or both have a modest growth impairment in fibroblasts [58]. Finally, one miRNA (miR-UL70-3p) was strongly induced in d-THP-1 cells but not in HELs, suggesting a possible role during macrophage infection but not in fibroblasts.

HCMV miRNA expression during quiescent infection of THP-1 cells was characterized by overall low level induction of all 16 miRNAs studied (Fig. S1). It therefore appears that miRNAs are subject to the same transcriptional repression that blocks IE expression in these cells. That none of the 16 miRNAs examined were robustly expressed within the first 24–48 hpi suggests that a role for these miRNAs in early suppression of the lytic program is unlikely. However, three miRNAs (miR-UL112, -UL70-3p, -US5-1) showed modest increases of 3- to 4-fold at 72 hpi, and thus could potentially function in maintenance of the quiescent state. That miR-US5-1 and miR-US5-2 target regulators of cell cycle during lytic replication may suggest a similar role during latency [15], [39].

Three previously uncharacterized miRNAs were tested for their ability to impact lytic replication when expressed ectopically (Fig. 4). Ectopic expression of miR-UL22A and miR-UL70 had no detectable effects on HCMV replication following infection at low or high MOI. However, ectopic expression was lower compared with levels induced by viral infection and may have been insufficient to induce a phenotype. Following low (0.01) MOI infection ectopically expressed miR-US33 impaired expression of IE, E, and late gene products, diminished accumulation of intracellular viral DNA, and reduced the yield of infectious progeny (Fig. 4). While these effects were modest and only observed at low MOI, it must again be considered that levels of ectopically expressed miR-US33 were much lower than those expressed during HCMV infection; thus, the impact of ectopic expression would likely only be manifested in the first 24–48 hpi, as by 48 hpi miR-US33 expressed from the HCMV genome would likely overwhelm that expressed ectopically.

Pre-miR-US33 is encoded by sequences complementary to *US29* and *US29* transcripts were therefore a potential miR-US33 target. The luciferase reporter assay confirmed the ability of miR-US33 to target proteins expressed from transcripts containing *US29* sequences and ectopic expression of miR-US33 reduced the level of viral *US29* mRNA in the context of HCMV infection (Fig. 5). While lack of a US29-specific antibody renders analysis of US29 protein levels currently impractical, the available data strongly suggest that US29 is a miR-US33 target. Based on growth properties of deletion mutants, *US29* has been classified as “dispensable” or “nonessential” for viral replication [59], [60]. However, these results do not preclude the possibility that US29 may have modest effects in augmenting viral replication as these studies considered mutants that replicated within 5-fold [60] or 10-fold [59] of wild type to be unimpaired. Thus, the impaired growth phenotype we observed in cells expressing ectopic miR-US33 could be a consequence of reduced US29 expression; however, the possibility that miR-US33 impairs HCMV replication by targeting other viral or cellular genes cannot presently be ruled out. Regardless of the target, the evidence indicates that miR-US33 represses lytic replication and thus could facilitate the establishment or maintenance of HCMV latency.

In summary, expression patterns for 16 HCMV miRNAs differed significantly following infection of cell culture systems representing permissive and semi-permissive lytic replication and quiescent genome maintenance. These differences in miRNA expression may imply diverse functional roles in different cellular environments and different viral replication programs. An additional HCMV miRNA (miR-US33) as found to suppress HCMV lytic replication, adding to an emerging theme that a significant subset of HCMV miRNAs function to down-modulate lytic replication. The role(s) such miRNAs may play in latency awaits further studies to characterize miRNA expression during natural latency and to evaluate miRNA null mutants in cell culture latency models.

## Supporting Information

Figure S1 **Expression kinetics of HCMV miRNAs during quiescent infection of THP-1 cells.** Undifferentiated THP-1 monocytes were infected with HCMV strain Towne at an MOI of 10 and intracellular HCMV miRNAs were quantitated by stem-loop RT-PCR at the indicated times post infection. Results indicate fold-changes relative to levels measured at 3 hpi.
(TIF)

Figure S2 **Expression kinetics of HCMV miRNAs during semi-permissive replication in d-THP-1 cells.** THP-1 monocytes were differentiated into macrophages by culture for 24 h in medium containing PMA and hydrocortisone. Resulting d-THP-1 cells were infected with HCMV strain Towne at an MOI of 10 and intracellular HCMV miRNAs were quantitated by stem-loop RT-PCR at the indicated times post infection. Results indicate fold-changes relative to levels measured at 3 hpi.
(TIF)

Table S1 **Comparison of Ct values of strongly induced miRNAs in d-THP-1 vs. HEL.**
(DOC)
