# Correction: Comprehensive Analysis of Human Cytomegalovirus MicroRNA Expression during Lytic and Quiescent Infection

**DOI:** 10.1371/journal.pone.0231909

**Published:** 2020-04-10

**Authors:** Zhang-Zhou Shen, Xing Pan, Ling-Feng Miao, Han-Qing Ye, Stéphane Chavanas, Christian Davrinche, Michael McVoy, Min-Hua Luo

After this article [1] was published, the authors noted a data reporting error in Figure 4B.

Specifically, the authors commented that the western blot images in Figure B were spliced between lanes 1 and 2 (mock and 24hpi/- lanes), where 4, 8, and 12 hpi timepoint data which showed no signal and no difference were removed from the images when preparing the figure. The lab has repeated the experiments and obtained consistent results. The original underlying data for these experiments are no longer available. Here, the authors provide an updated version of Figure 4B in which the western blot panels are replaced with replication data, along with the underlying image data for the reported replication experiments (S1 File).

A member of *PLOS ONE*’s Editorial Board reviewed the updated figure and supporting data and confirmed that they support the results reported in the original article.

The data underlying other results reported in the article are available upon request.

The authors apologize for the errors in the original figure.

*PLOS ONE* apologizes for the delay in posting this Correction, and acknowledges that the authors responded to the concerns about this article by providing data and clarifications in 2017.

## Supporting information

S1 File Updated version of Figure 4B reporting results of replication experiments, and the original blot images supporting the updated figure panel. HELs were infected with HCMV strain Towne at a MOI of 0.01 at 48 h after transduction with control lentivirus or lentivirus expressing the indicated miRNAs. Cells were harvested at the indicated times, and levels of IE1, IE2, UL44, or gB were determined by western blotting. Actin serves as a loading control. Control lentivirus (-) or lentiviruses expressing according miRNAs (+) are indicated.
(TIF)
