# Supplementary figures and images for: Comprehensive Analysis of Human Cytomegalovirus MicroRNA Expression during Lytic and Quiescent Infection
Source: PLoS One. 2014 Feb 12;9(2):e88531. doi: 10.1371/journal.pone.0088531 (PMC3922878; doi:10.1371/journal.pone.0088531)

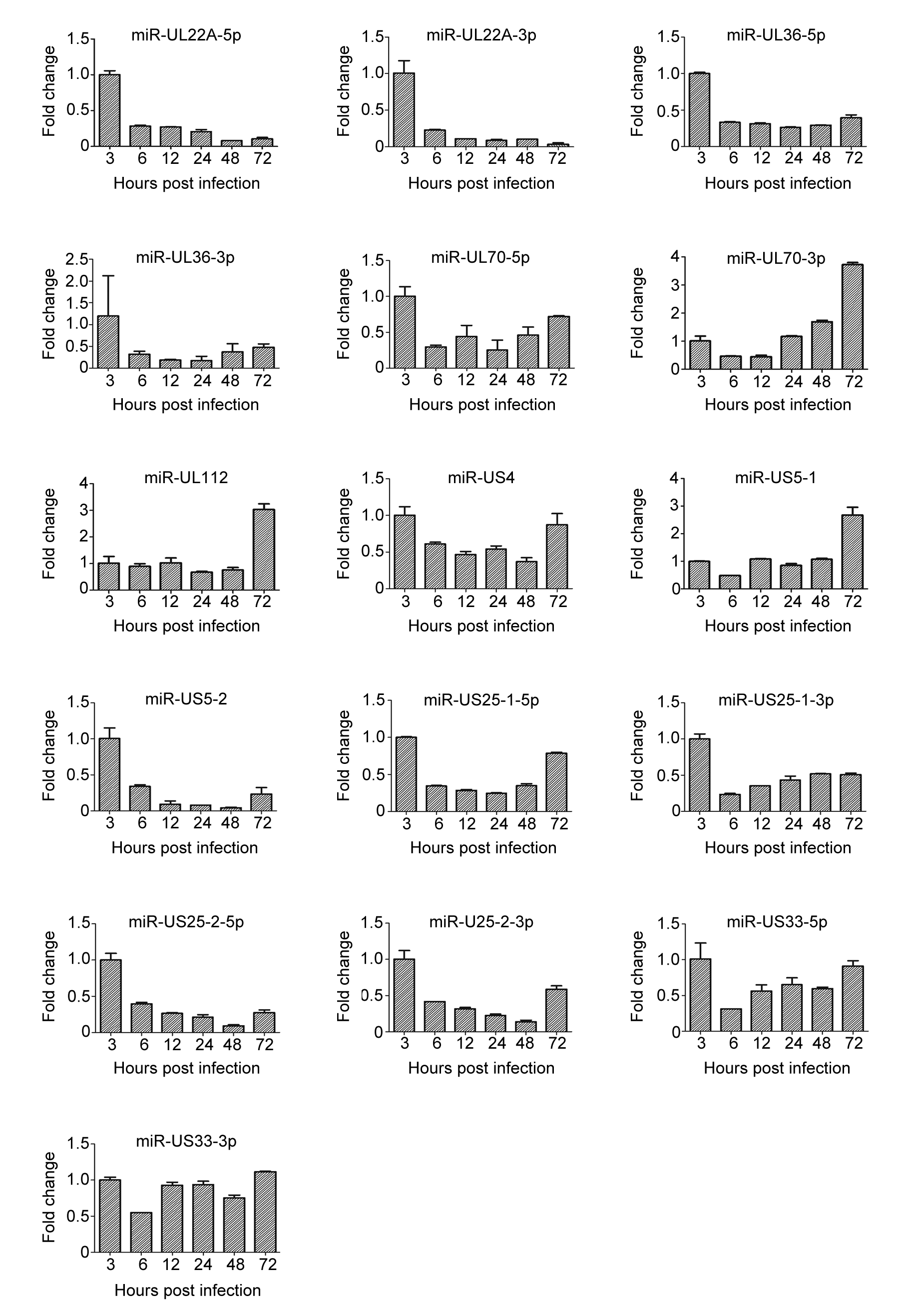

Supplement: Figure S1 — Expression kinetics of HCMV miRNAs during quiescent infection of THP-1 cells. Undifferentiated THP-1 monocytes were infected with HCMV strain Towne at an MOI of 10 and intracellular HCMV miRNAs were quantitated by stem-loop RT-PCR at the indicated times post infection. Results indicate fold-changes relative to levels measured at 3 hpi. (TIF) [file pone.0088531.s001.tif]

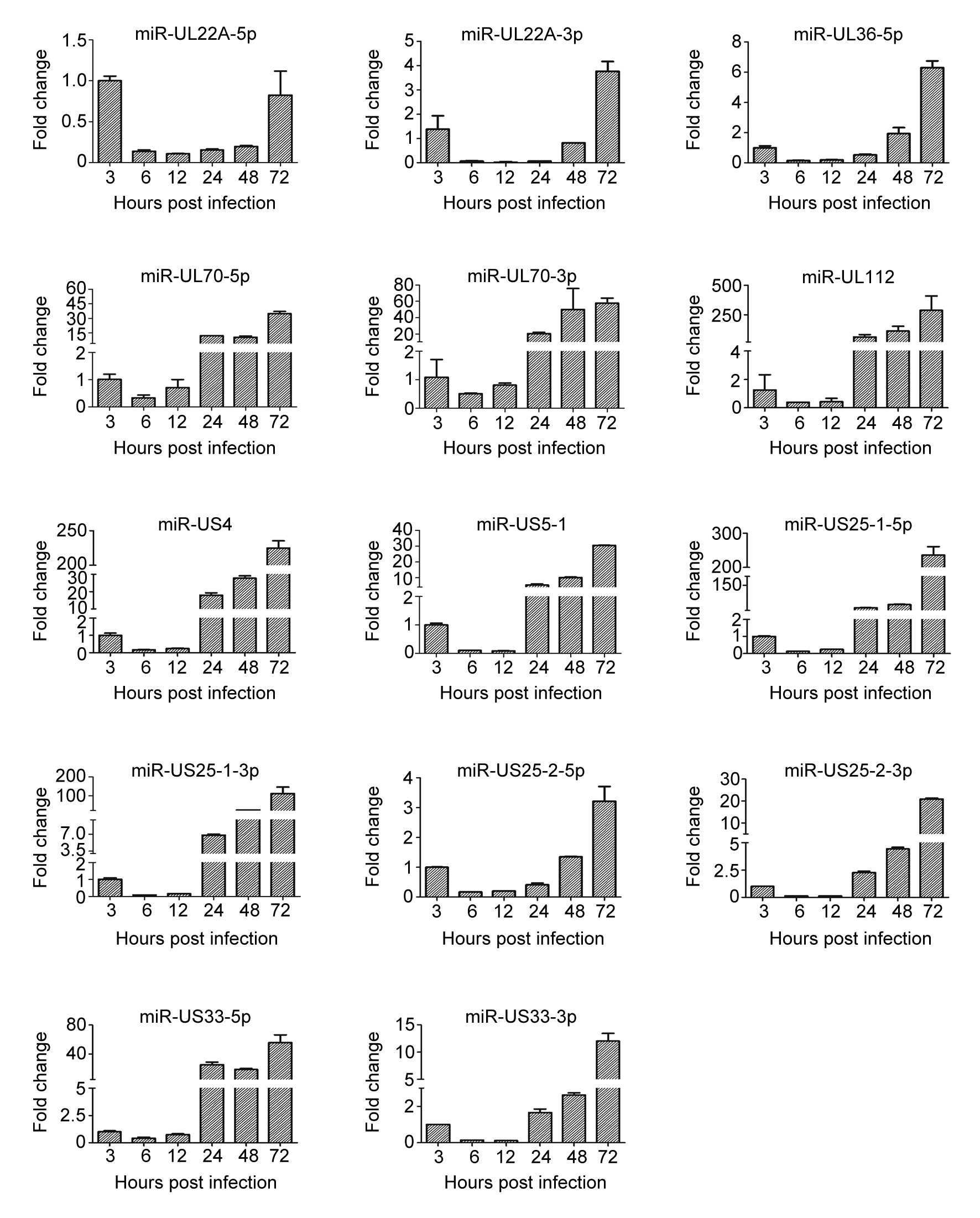

Supplement: Figure S2 — Expression kinetics of HCMV miRNAs during semi-permissive replication in d-THP-1 cells. THP-1 monocytes were differentiated into macrophages by culture for 24 h in medium containing PMA and hydrocortisone. Resulting d-THP-1 cells were infected with HCMV strain Towne at an MOI of 10 and intracellular HCMV miRNAs were quantitated by stem-loop RT-PCR at the indicated times post infection. Results indicate fold-changes relative to levels measured at 3 hpi. (TIF) [file pone.0088531.s002.tif]
