# Supplementary material for: Comprehensive Analysis of Human Cytomegalovirus MicroRNA Expression during Lytic and Quiescent Infection
Source: PLoS One. 2014 Feb 12;9(2):e88531. doi: 10.1371/journal.pone.0088531 (PMC3922878; doi:10.1371/journal.pone.0088531)
Supplement: Table S1 — Comparison of Ct values of strongly induced miRNAs in d-THP-1 vs. HEL. (DOC) [file pone.0088531.s003.doc]

Table S1. Comparison of Ct values of strongly induced miRNAs in d-THP-1 vs. HEL

| miRNAs | Ct value | | Level | |
| --- | --- | --- | --- | --- |
|  | d-THP-1 | HELs | d-THP-1 | HELs |
| miR-UL70-3p | 19.86 | 19.42 | 1 | 2.45 |
| miR-UL112 | 24.55 | 21.22 | 1 | 11.99 |
| miR-US4 | 24.12 | 26.43 | 1 | 0.13 |
| miR-US25-1-5p | 17.84 | 19.86 | 1 | 0.13 |
| miR-US25-1-3p | 28.25 | 28.08 | 1 | 1.56 |
| miR-US33-5p | 31.24 | 31.44 | 1 | 0.6 |
