# Supplementary figures and images for: Correction: Comprehensive Analysis of Human Cytomegalovirus MicroRNA Expression during Lytic and Quiescent Infection
Source: PLoS One. 2020 Apr 10;15(4):e0231909. doi: 10.1371/journal.pone.0231909 (PMC7147750; doi:10.1371/journal.pone.0231909)

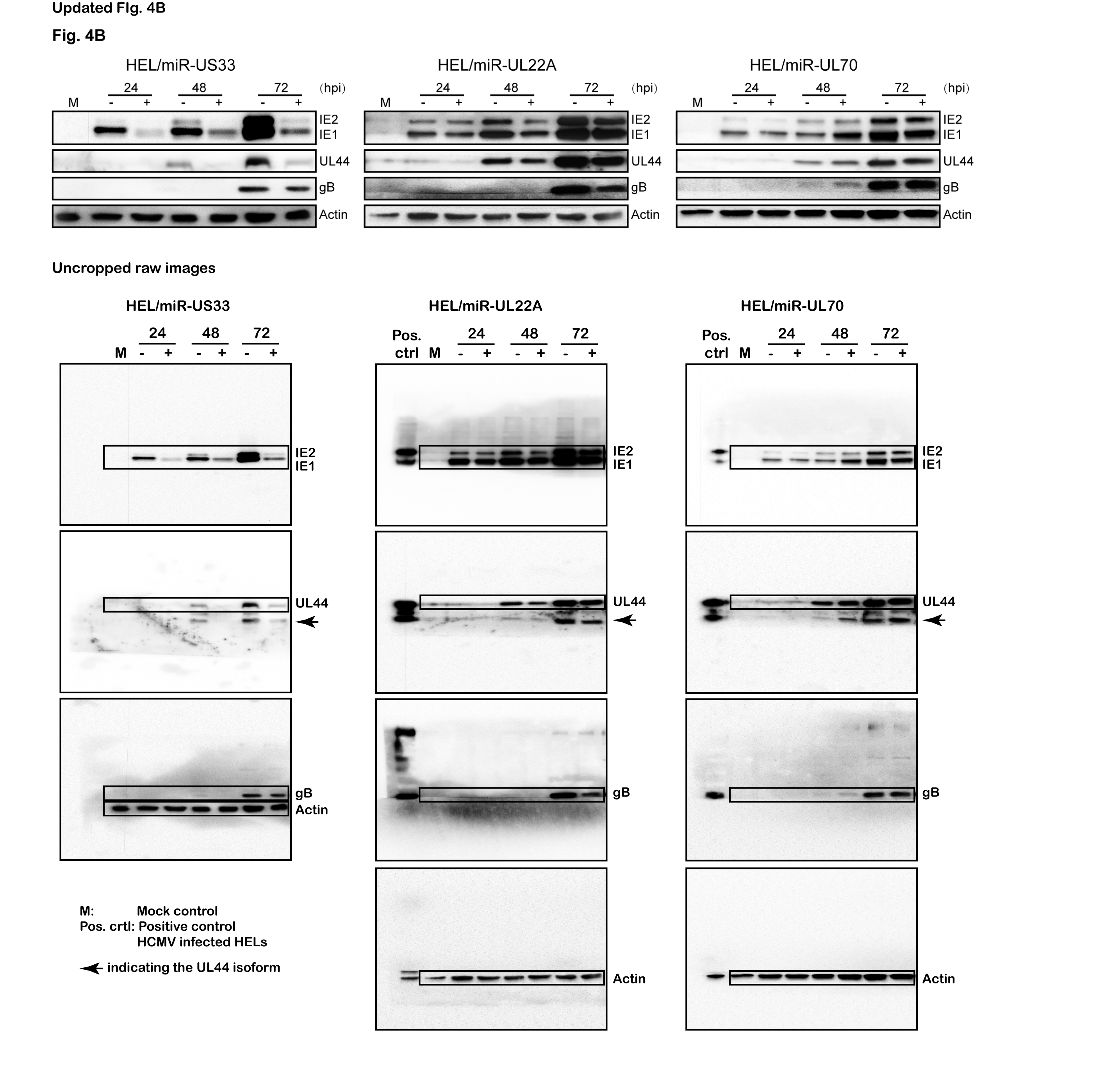

Supplement: S1 File — HELs were infected with HCMV strain Towne at a MOI of 0.01 at 48 h after transduction with control lentivirus or lentivirus expressing the indicated miRNAs. Cells were harvested at the indicated times, and levels of IE1, IE2, UL44, or gB were determined by western blotting. Actin serves as a loading control. Control lentivirus (-) or lentiviruses expressing according miRNAs (+) are indicated. (TIF) [file pone.0231909.s001.tif]
